# Genotype-by-environment interaction of fertility traits in Danish Holstein cattle using a single-step genomic reaction norm model

**DOI:** 10.1038/s41437-019-0192-4

**Published:** 2019-02-13

**Authors:** Zhe Zhang, Morten Kargo, Aoxing Liu, Jørn Rind Thomasen, Yuchun Pan, Guosheng Su

**Affiliations:** 1 Department of Molecular Biology and Genetics, Center for Quantitative Genetics and Genomics, Aarhus University, Blichers Allé 20, Postboks 50, Tjele, DK 8830 Denmark; 2 Department of Animal Science, School of Agriculture and Biology, Shanghai Jiao Tong University, 200240 Shanghai, China; 3 Knowledge Center for Agriculture (SEGES), Skejby, Aarhus N, 8200 Denmark; 4 College of Animal Science and Technology, China Agricultural University, Beijing, 100193 China; 5 VikingGenetics, Asesentoft, 8960 Denmark

**Keywords:** Animal breeding, Genome-wide association studies

## Abstract

Genotype-by-environment (G × E) interactions could play an important role in cattle populations, and it should be considered in breeding programmes to select the best sires for different environments. The objectives of this study were to study G × E interactions for female fertility traits in the Danish Holstein dairy cattle population using a reaction norm model (RNM), and to detect the particular genomic regions contributing to the performance of these traits and the G × E interactions. In total 4534 bulls were genotyped by an Illumina BovineSNP50 BeadChip. An RNM with a pedigree-based relationship matrix and a pedigree-genomic combined relationship matrix was used to explore the existence of G × E interactions. In the RNM, the environmental gradient (EG) was defined as herd effect. Further, the genomic regions affecting interval from calving to first insemination (ICF) and interval from first to last insemination (IFL) were detected using single-step genome-wide association study (ssGWAS). The genetic correlations between extreme EGs indicated that G × E interactions were sizable for ICF and IFL. The genomic RNM (pedigree-genomic combined relationship matrix) had higher prediction accuracy than the conventional RNM (pedigree-based relationship matrix). The top genomic regions affecting the slope of the reaction norm included immunity-related genes (*IL17*, *IL17F* and *LIF*), and growth-related genes (*MC4R* and *LEP*), while the top regions influencing the intercept of the reaction norm included fertility-related genes such as *EREG*, *AREG* and *SMAD4*. In conclusion, our findings validated the G × E interactions for fertility traits across different herds and were helpful in understanding the genetic background of G × E interactions for these traits.

## Introduction

In the dairy cattle industry, fertility traits are some of the most influential components, as declining fertility prolongs the resume cycles after calving and increases veterinary costs (De Vries 2006; Schneider et al. 2005). Some previous studies have emphasized the importance of genetic evaluation and the improvement of fertility traits in spite of their low heritability (below 5%) (Liu et al. 2017; Sun et al. 2010), and more balanced selection indices including production, longevity, health and fertility have been used instead of indices which simply focused on yield. Another issue regarding selection for improved fertility is that a wide range of environments often contribute to the phenomenon of genotype-by-environment (G × E) interaction, which is defined as different performances of animals and their offspring in different environments than those where they were raised or selected (Falconer et al. 1996).

In the Danish milk production system, different herds have different conditions in terms of feed, pharmaceuticals and housing, which may further lead to G × E interactions (Strandberg et al. 2000), and can therefore be defined as an environmental gradient (EG) to study the G × E interactions. Two models are widely used to detect G × E interactions. One is a multi-trait model, which assumes that phenotypic expressions of a trait in various environments are different traits (Kolmodin et al. 2002), and a low genetic correlation among these different traits indicates the existence of a G × E interaction. The other one is the reaction norm model (RNM) (Falconer et al. 1996), which models the trajectory of animal performance as a function of the EG, and the breeding value of an animal is therefore partitioned into an environment-independent part (intercept) and an environment-dependent (slope) part. Compared with multi-trait models, RNMs are able to explore G × E interactions in a range of continuous environments and quantify the G × E interaction at any environment within the range.

The application of an RNM often uses the known EG as a covariate. Traditionally, the covariate was postulated as the phenotypic mean in different environments, which could lead to bias due to misleading representation of EG. An alternative method which infers the unknown EG using the Bayesian method, was suggested, and more accurate estimates could be obtained (Su et al. 2006, 2009). Although the Bayesian method is able to handle the unknown EG, the analysis is time consuming, and sometimes it is difficult to get model convergence when the model becomes complicated and too many parameters need to be estimated at the same time. Calus et al. (2002) and Kolmodin et al. (2002) proposed to first estimate the EG using a conventional linear mixed model and substitute it into the RNM to study the G × E interaction, which can be viewed as partitioning the simultaneous estimation of the EG and other parameters into two steps.

Furthermore, the inclusion of genotype information in the RNM may be beneficial for the estimation of parameters and prediction of breeding values. To integrate information from genotyped and non-genotyped animals, a single-step genomic BLUP (ssGBLUP) (Christensen and Lund 2010; Misztal et al. 2009) can be used in complicated models such as random regression or RNMs. In addition, Wang et al. 2012 proposed a new method to perform genome-wide association study (GWAS) based on ssGBLUP, which is called a single-step GWAS (ssGWAS). A simulation study has shown that this method results in more accurate estimates of SNP effects compared to BayesB and the conventional GWAS methods with single-marker regression.

The objective of this study was to use the RNM with information on genomic markers and pedigrees to explore the G × E interaction of female fertility for Danish Holstein dairy cows and to map the genomic regions contributing to the fertility phenotypes across different EGs.

## Materials and methods

### Data

The fertility traits of Danish Holstein used in this study included number of inseminations per conception (AIS), interval from first to last insemination (IFL), non-return rate at 56 days after first insemination (NRR) and interval from calving to first insemination (ICF). Field records during the period of insemination from 2011 to 2016 were collected from 1775 herds. The records in the first three parities were used in this study.

In addition, AIS and IFL included some censored records because of no known date of confirmed successful insemination. In this study, penalty values were used to handle censored data (Liu et al, 2017; Sun et al, 2009). For censored IFL, a penalty of 40 days was added, which is the average value of Danish Holstein cows, and a penalty of 1 was added to censored AIS. The further criteria for the data editing were in accordance with Nordic routine genetic evaluation. Briefly, the criteria included age at first insemination between 270 and 900 days, age at first calving between 500 and 1100 days, AIS between 1 and 5, IFL between 0 and 365 days, ICF between 20 and 230 days, and days open (ICF + IFL) between 20 and 365 days. Records with values below the lower limit were removed, whereas records were converted to upper-limit values if they were greater than the upper limit. Herds were defined as the herds where the animals got phenotypes. Animals that moved to other herds between the first insemination and the last insemination were removed. In addition, for each trait, the herd-year levels with <20 records were merged with the nearest herd-year level, and this combination was performed once more if the number of records was still <20. Finally, the records in herd-year levels with <20 records were discarded after two combination runs. The detailed data editing procedure can be found online (https://www.nordicebv.info/).

The number of records and animals for each trait obtained after editing are listed in Table 1. The pedigree of the animals with phenotypes was traced three generations back using the Nordic Cattle database (NAV, Skejby, Denmark).

**Table 1:** Descriptive statistics for female fertility traits and heritabilities (standard errors) estimated using a pedigree-based linear mixed model

Trait	No. of records	No. of animals	Mean	SD	Min	Max	Heritability
AIS	705,835	383,179	2.06	1.24	1	5	0.027 (0.001)
ICF	723,333	391,930	74.4	31.1	20	180	0.057 (0.003)
IFL	728,426	393,561	47.7	61.3	0	230	0.032 (0.002)
NRR	715,911	386,869	0.542	0.498	0	1	0.011 (0.001)

*AIS* number of inseminations per conception, *ICF* interval from calving to first insemination, *IFL* interval from first to last insemination, *NRR* non-return rate at 56 days after first insemination, *SD* standard deviation, *Min* minimum value, *Max* maximum value

Genotypes were available for 4534 bulls in the pedigree, among which 1777 bulls have daughters with records in the dataset. The bulls were genotyped using an Illumina BovineSNP50 BeadChip (Illumina Inc., San Diego, CA, USA) version 2 (containing 54,609 single nucleotide polymorphisms (SNPs)). The SNP data were filtered by removing markers with a minor allele frequency <1%, an average GenCall score lower than 0.60, or an unknown location in the UMD 3.1 assembly. After data editing, 46,342 SNPs were included in the analysis.

### Model

The EG defined in this study included the production environments of herds, i.e., herd effects. We used a three-step strategy to analyze the G × E interaction for different traits. This three-step approach is an extension of the two-step RNM proposed by Calus et al. (2002) and Kolmodin et al. (2002). Briefly, the first two steps were designed to estimate herd effects and weights of heterogeneous residual variances in RNM of the third step, respectively, so that the RNM was simplified. Compared with the one-step method that estimates an unknown EG and other parameters simultaneously using a Bayesian method (Su et al. 2006), the three-step approach has much less computational demand.

### Step 1

Initially, a univariate animal model was used to fit observations for each trait. The model was as follows:

1 $${\boldsymbol{y}}{\mathrm{ = }}{\boldsymbol{Xb}} + {\boldsymbol{Ff}} + {\boldsymbol{Za}} + {\boldsymbol{Ec}} + {\boldsymbol{Wpe}} + {\boldsymbol{e}}$$

where ***y*** is the vector of observations; ***b*** is the vector of fixed effects other than herd effect; ***f*** is the vector of fixed herd effects; ***a*** is the vector of additive genetic effects, which are assumed to follow the normal distribution $N\left( {0,\,{\boldsymbol{A}}\sigma _a^2} \right)$ for a pedigree-based BLUP or $N\left( {0,\,{\boldsymbol{H}}\sigma _a^2} \right)$ for an ssGBLUP model, where $\sigma _a^2$ is the additive genetic variance and ***A*** is the numerator (pedigree) relationship matrix and ***H*** is the combined pedigree-genomic relationship matrix; ***c*** is the vector of herd-year effects, which are assumed to be normally distributed with $N\left( {0,\,{\boldsymbol{I}}\sigma _c^2} \right)$, where $\sigma _c^2$ is the variance of herd-year effects and ***I*** is the identity matrix**;** ***pe*** is the vector of permanent environmental effects, which are assumed to be normally distributed with $N( {0,\,{\boldsymbol{I}}\sigma _{pe}^2} )$, where $\sigma _{pe}^2$ is the variance of permanent environmental eff***e***cts; ***e*** is the random residual vector following $N\left( {0,\,{\boldsymbol{I}}\sigma _e^2} \right)$ and $\sigma _e^2$ is the random residual variance. ***X***, ***F***, ***Z***, ***E*** and ***W*** and are incidence matrices related to the fixed and random effects in the model. The fixed effects included herd, parity, year of first insemination (for AIS, IFL and NRR) or year of calving (for ICF), month of first insemination (for AIS, IFL and NRR) or month of calving (for ICF) and age at first insemination (divided into five age groups of 270–419 days, 420–444 days, 445–469 days, 470–500 days and 500–900 days). The random effects included additive genetic effects, herd-year effects (year of first insemination for AIS, IFL and NRR; year of calving for ICF), and permanent effects.

For the pedigree-based BLUP, the numerator relationship matrix (***A***) was built based only on the pedigree, whereas for the ssGBLUP model, a combined pedigree-genomic relationship matrix (***H***) was used, and the inverse ***H−***^1^ was calculated as follows (Aguilar et al. 2010; Christensen and Lund 2010):

2 $${\boldsymbol{H}}^{ - 1} = {\boldsymbol{A}}^{ - 1} + \left[ {\begin{array}{*{20}{c}} 0 & 0 \\ 0 & {{\boldsymbol{G}}^{ - 1} - {\boldsymbol{A}}_{22}} \end{array}} \right]$$

where ***A***_22_ is the subset of the numerator relationship matrix for genotyped animals. ***G*** is the blended genomic relationship matrix. ***G*** was constructed as (1−ω)***G***_0_+ω***A***_22_, where ω is the relative weight that could explain the fraction of the genetic variance not captured by markers and was set as 0.2 according to (Gao et al. 2012); ***G***_0_ (VanRaden 2008) was constructed using Gmatrix software (Su and Madsen 2011):

3 $${\boldsymbol{G}}_0 = {\boldsymbol{ZDZ}}\prime /\mathop {\sum}\nolimits_{i = 1}^m {2p_i\left( {1 - p_i} \right)}$$

where the elements in column *i* of ***Z*** are 0−2_*pi*_, 1−2_*pi*_, and 2−2_*pi*_ for genotypes A_1_A_1_, A_1_A_2_ and A_2_A_2_, respectively, where _*pi*_ is the allele frequency of A_2_ at locus *i* calculated from the current data; ***D*** is a diagonal weight matrix for each SNP, and in this step, ***D*** is an identity matrix; .*m* is the number of SNPs. At last, ***G*** was adjusted to be compatible with **A**_22_ according to Christensen et al. 2012. The analysis was performed using the DMUAI module implemented in the DMU package (Madsen et al. 2013).

### Step 2

In an RNM, heterogeneous residual variances are usually fitted when residual variances differ among production environments (Oliveira et al. 2018; Silva et al. 2014). To account for various residual variances, the herds were divided into five groups according to the quantiles of the herd effects estimated in step 1, namely, (0, 10%), (10, 30%), (30, 70%), (70, 90%), and (90, 100%). The setting of five groups was based on a balance between number of observations in each group and the possible difference in residual variance among different herd levels. Then, the model in step 1 was re-run, and the variance components were estimated by a Gibbs sampling approach using the RJMC module in the DMU package (Madsen et al. 2013).

### Step 3

Compared with conventional linear mixed model, the RNM included genetic sensitivities represented by random regressions on the herd effects estimated in the step 1 and was analyzed using DMUAI. It was shown as follows:

4 $${\boldsymbol{y}} = {\boldsymbol{Xb}} + {\boldsymbol{Ff}} + {\boldsymbol{Z}}_0{\boldsymbol{a}}_0 + {\boldsymbol{Z}}_1{\boldsymbol{a}}_1 + {\boldsymbol{Ec}} + {\boldsymbol{Wpe}} + {\boldsymbol{e}}$$

where it is assumed that $$\left[ {\begin{array}{*{20}{c}} {{\boldsymbol{a}}_0} \\ {\begin{array}{*{20}{c}} {{\boldsymbol{a}}_1} \end{array}} \end{array}} \right]\sim {\boldsymbol{N}}\left( {0,\,{\boldsymbol{A}} \otimes \left[ {\begin{array}{*{20}{c}} {\sigma _{a_0}^2} & {\sigma _{a_0a_1}} \\ {\sigma _{a_0a_1}} & {\sigma _{a_1}^2} \end{array}} \right]} \right)$$. ***Z***_0_ is an incidence matrix connecting ***a***_0_ (intercept) to ***y***, and ***Z***_1_ is an incidence matrix containing herd effects estimated in step 1 as covariables to connect ***y*** and ***a***_1_ (slope). Other effects and incidence matrices are the same as those for linear mixed models in step 1. The random residual vector is assumed to follow $N\left( {0,\,{\boldsymbol{R}}\sigma _e^2} \right)$, where ***R*** is a diagonal matrix with elements for observations in the *i*th group of herds equal to 1/*w*_*i*_, and

5 $$w_i = \frac{{\mathop {\sum }\nolimits_{i = 1}^5 n_i\sigma _{e_i}^2}}{{\sigma _{e_i}^2\mathop {\sum }\nolimits_{i = 1}^5 n_i}}$$

where *n*_*i*_ is the number of observations in the *i*th group of herds and $\sigma _{e_i}^2$ is the residual variance of the *i*th group estimated by RJMC. In addition, to evaluate the need to include the G × E interaction in the model, a likelihood ratio test (LRT) between the RNM and the reduced model (RM) of (4) without considering random regression on herd effects was performed based on the statistic of *D* = −2 * log(likelihood) for the RM, + 2 * log(likelihood) for the RNM. The *P*-value for the LRT was calculated as $0.5{\mathrm{P}}\left[ {{\mathrm{\chi }}_{1\,d.f.}^2 \ge {D}} \right] + 0.5{\mathrm{P}}\left[ {{\mathrm{\chi }}_{2\,d.f.}^2 \ge {D}} \right]$.

The heritability $\left( {h_a^2} \right)$ of the linear mixed model was calculated as $h_a^2 = \frac{{\sigma _a^2}}{{\sigma _a^2 + \sigma _{pe}^2 + \sigma _e^2}}$. The additive genetic variances $\left( {\sigma _a^2} \right)$ and heritability $\left( {h_a^2} \right)$ of the RNM with a particular herd effect (*f*) were calculated as $\sigma _a^2 = \sigma _{a_0}^2 + \sigma _{a_1}^2f^2 + 2\sigma _{a_0a_1}f$. The residual variance $\left( {\sigma _e^{ \ast 2}} \right)$ of the RNM in a particular herd group was calculated as $\sigma _e^{ \ast 2} = \frac{1}{w}\sigma _e^2$ . Consequently, the heritability $\left( {h_a^2} \right)$ of the RNM was calculated as $h_\alpha ^2 = \frac{{\sigma _\alpha ^2}}{{\sigma _\alpha ^2 + \sigma _{pe}^2 + \sigma _e^{ \ast 2}}}$. Standard errors of heritabilities and genetic correlations were calculated according to an expansion of the Taylor series (Su et al. 2007).

### Prediction of future performances

To evaluate and compare the prediction ability of different models, the data were divided into training and validation parts according to the birthdates of the bulls. The 20% youngest bulls (born after 01/07/2010, 355 bulls) with daughter information were regarded as the validation dataset, and the other 80% were regarded as the training dataset. In the RNM, the estimated breeding value of a particular bull obtained from the pedigree-based BLUP (EBV) or from the ssGBLUP (GEBV) was defined as

6 $$\widehat a = \widehat a_0 + \overline f \widehat a_1$$

where $\widehat a$ is the predicted breeding value for this bull; $\widehat a_0$ and $\widehat a_1$ are estimates of the intercept and slope for this bull, respectively; $\overline f$ is the mean effect of herds where daughters of this bull were raised. Daughter yield deviation (DYD) was used to evaluate the accuracies of the prediction. The DYD was the average performance of daughters adjusted for all fixed and non-genetic random effects as well as the dam’s additive genetic effect. The accuracy of the prediction was calculated as a Pearson correlation in the form of cor(DYD, prediction). In addition, the accuracy of the prediction obtained from the RNM was compared to that calculated from the RM.

### ssGWAS for ICF and IFL

ssGWAS as proposed by Wang et al. 2012 was applied to locate the genomic regions related to the intercept and slope in the RNM for ICF and IFL, as it was observed that there existed significant G × E interactions across extreme EGs, and slightly higher prediction accuracies for the RNM compared to the RM for the two traits. In a typical ssGBLUP model, it is assumed that all SNP effects have the same variance. The ssGWAS allows heterogeneous variance among different SNPs, which is realized by a weighted G-matrix, and the weights can be obtained by an iteration procedure. Thus, SNP effects and weights for ssGWAS were calculated as follows:

1. Calculate the ***G*** matrix with *D*_*ii*_ = 1 using (3).

2. Calculate the GEBV for all the genotyped animals using the reaction norm based ssGBLUP.

3. Calculate the SNP effects $\left( {\widehat {\boldsymbol{u}}} \right)$ based on the GEBV with $\widehat {\boldsymbol{u}} = {\boldsymbol{DZ}}\prime \left( {{\boldsymbol{ZDZ}}\prime } \right)^{ - 1}\widehat {\boldsymbol{a}}$, where $\widehat {\boldsymbol{a}}$ is the vector of the GEBV with elements calculated using formula (6).

4. Calculate the weight (*D*_*ii*_) for each SNP as $D_{ii} = \widehat u_i^22p_i\left( {1 - p_i} \right)$, where _*pi*_ is the allele frequency for the *i*th SNP.

5. Calculate ***D*** by normalizing the SNP weights to keep the total genetic variance constant.

6. Repeat steps 2–5.

Considering that too many iterations may cause some subjective peaks, the ssGWAS was run for three iterations (Wu et al. 2018), and in the last iteration, the SNP effects for the intercept $\left( {\widehat {\boldsymbol{u}}_0} \right)$ and slope $\left( {\widehat {\boldsymbol{u}}_1} \right)$ were calculated as $\widehat {\boldsymbol{u}}_{\boldsymbol{s}} = {\boldsymbol{DZ}}\prime \left( {{\boldsymbol{ZDZ}}\prime } \right)^{ - 1}\widehat {\boldsymbol{a}}_s$, where *s* equals 0 for the intercept and 1 for the slope. The percentage of genetic variances of the slope or intercept explained by a discrete window of 20 adjacent SNPs was defined as $\frac{{Var\left( {a_{st}} \right)}}{{\sigma _{a_s}^2}} \times 100\%$, where *a*_*st*_ is the genetic value for the intercept (*s* = 0) or slope (*s* = 1) of the *t*th window and can be calculated as $\mathop {\sum}\nolimits_{k = 1}^{20} {{\boldsymbol{Z}}_k\widehat u_{sk}}$, where ***Z***_*k*_ is the matrix of gene content of the *k*th SNP for genotyped individuals, and $\widehat u_{sk}$ is the SNP effect of the *k*th SNP with the *t*th window for the intercept or slope; $\sigma _{a_s}^2$ is the total additive genetic variance for the intercept or slope. In addition, the remaining SNPs (<20) at the end of the chromosome were merged into the last window.

Candidate genes within the top 10 windows with the highest percentages of genetic variances of the intercept or slope were retrieved by the *BioMart* package (Durinck et al. 2005) based on UMD 3.1 assembly.

## Results

### Descriptive statistics

The summary statistics for each trait are listed in Table 1. A large amount of phenotypic variation was observed for each trait. Table 1 also lists the heritability of each trait estimated by the conventional linear mixed model based on pedigree information. As expected, the heritability estimates for these fertility traits were quite low, which is consistent with an earlier study (Liu et al. 2017).

### Variance and covariance components estimated by RNM

The estimates of variance components and their standard errors obtained by the RNMs based on different relationship matrices (***A*** and ***H*)** were very similar (Table 2). Table 2 also lists the correlation coefficients between the intercept and slope for each trait. All of these correlation coefficients were negative and ranged from −0.880 to −0.360.

**Table 2:** Variance of the intercept $( {\sigma _{a_0}^2} )$, variance of the slope $( {\sigma _{a_1}^2} )$, covariance between the intercept and slope $( {\sigma _{a_0a_1}} )$, permanent environmental variance $( {\sigma _{pe}^2} )$, variance of herd-year effects $( {\sigma _{hy}^2} )$, residual variance $( {\sigma _{hy}^2} )$ and correlation between the intercept and slope $( {r_{a_0a_1}} )$, with their standard errors in parentheses estimated using an RNM

Trait	*A*|*H*	$\sigma _{a_0}^2$	$\sigma _{a_1}^2$	$\sigma _{a_0a_1}$	$\sigma _{pe}^2$	$\sigma _{hy}^2$	$\sigma _e^2$	$r_{a_0a_1}$
AIS	***A***	0.083 (0.025)	0.046 (0.009)	−0.054 (0.015)	0.052 (0.003)	0.052 (0.003)	1.40 (0.003)	−0.880 (0.030)
	***H***	0.076 (0.024)	0.044 (0.009)	−0.050 (0.014)	0.052 (0.003)	0.010 (0.0005)	1.40 (0.003)	−0.871 (0.033)
ICF	***A***	92.0 (15.8)	0.015 (0.003)	−0.849 (0.193)	53.3 (2.05)	38.7 (0.930)	728.1 (1.70)	−0.724 (0.047)
	***H***	95.5 (15.9)	0.016 (0.003)	−0.883 (0.195)	52.1 (2.10)	38.7 (0.929)	728.2 (1.70)	−0.725 (0.046)
IFL	***A***	48.0 (22.0)	0.052 (0.011)	−0.621 (0.462)	168.2 (7.76)	18.3 (1.03)	3383.1 (8.03)	−0.395 (0.178)
	***H***	44.7 (21.3)	0.050 (0.011)	−0.535 (0.446)	166.6 (7.85)	18.3 (1.03)	3383.9 (8.03)	−0.360 (0.192)
NRR	***A***	0.006 (0.002)	0.009 (0.005)	−0.005 (0.003)	0.004 (0.0004)	0.001 (0.00007)	0.235 (0.0005)	−0.709 (0.116)
	***H***	0.005 (0.0017)	0.009 (0.005)	−0.005 (0.003)	0.004 (0.0004)	0.001 (0.00007)	0.235 (0.0005)	−0.690 (0.122)

*AIS* number of inseminations per conception, *ICF* interval from calving to first insemination, *IFL* interval from first to last insemination, *NRR* non-return rate at 56 days after first insemination, ***A*** and ***H*** pedigree-based and pedigree-genomic combined matrices, respectively

**Fig. 1:**
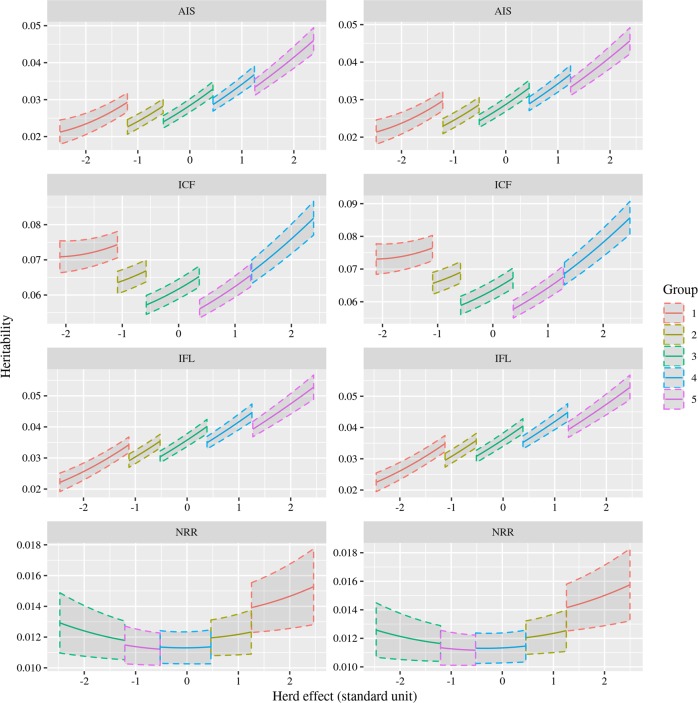
Heritabilities estimated using an RNM with the ***A*** relationship matrix (left) and ***H*** relationship matrix (right) for female fertility traits: AIS (number of inseminations per conception), ICF (interval from calving to first insemination), IFL (interval from first to last insemination), and NRR (non-return rate at 56 days after first insemination). The *x*-axis represents the normalized herd effects with a range of mean ± 2.5 standard units. The lines with different colours show the heritabilities within different groups of heterogeneous residual variance along different EGs, and the shades represent standard errors

The heritabilities among different herd levels (in the range of mean ± 2.5 × standard deviation of herd effects) estimated by the RNM based on the ***A*** matrix and those from the RNM based on the ***H*** matrix were similar (Fig. 1). The highest heritabilities for these traits were generally observed in herds with the largest effects. The least amount of variation in heritibilities among herd levels was observed for NRR, but with large standard errors. Figure 1 also shows the discrete lines of heritabilities along the continuous EGs, which were caused by the heterogeneous residual variances assumed for five groups of herds in the model. Generally, the larger the herd effects were, the larger the residual variance was and thus the smaller weights assigned to the residual variance were, although this was not the case for NRR and ICF (Table S1).

### Environmental sensitivity

**Fig. 2:**
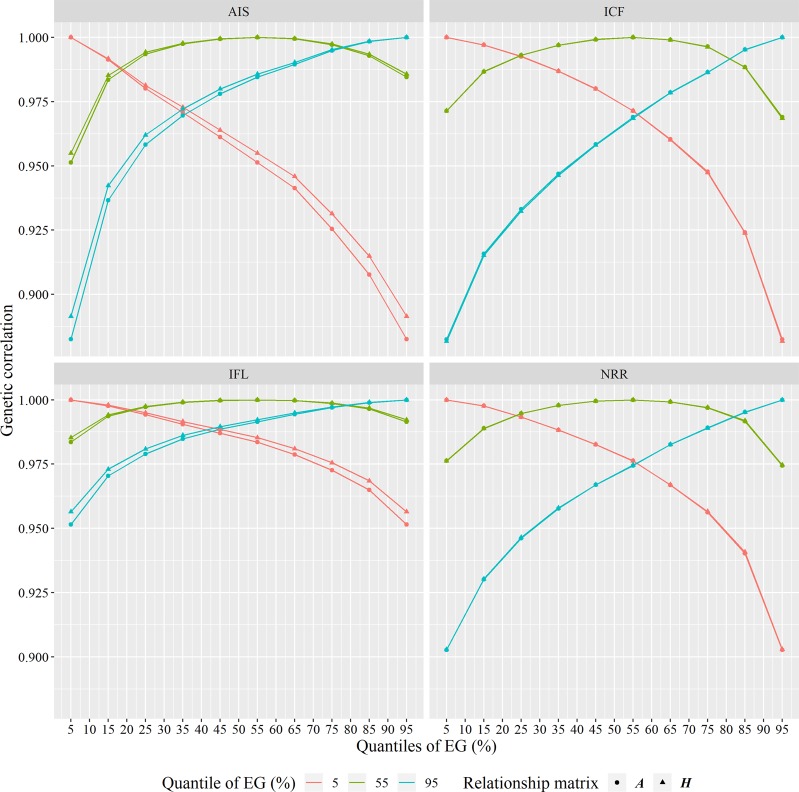
The genetic correlations between performances in a herd at a particular quantile of environmental gradients (EGs) and a herd at 5, 55 or 95% quantile of EGs for AIS (number of inseminations per conception), ICF (interval from calving to first insemination), IFL (interval from first to last insemination), and NRR (non-return rate at 56 days after first insemination) obtained from an RNM with the ***A*** relationship matrix and ***H*** relationship matrix. The x-axis represents different quantiles of EGs with a range of mean ± 2.5 standard units, and y-axis represents genetic correlation

The genetic sensitivity to different environments for each trait was indicated by the variance of the slope (Table 2), which was statistically (*P*-values < 0.05) different from zero for all traits based on one-tailed *t*-tests. The genetic correlations between a herd at a particular quantile of herd effects (in the range of mean ± 2.5 × standard deviation of herd effects) and herd level at 5% (lowest), 55% (intermediate) or 95% (highest) quantiles are shown in Fig. 2. The differences of patterns between ***A*** and ***H*** are very small for ICF and NRR, while the genetic correlations estimated by ***A*** matrix were slightly lower than those estimated by ***H*** matrix for AIS and IFL (Fig. 2). The values of genetic correlations between extreme EGs are listed in Table 3. It was observed that genetic correlations between quantiles of (1 and 99%), (5 and 95%), (10 and 90%) and (20 and 80%) of herd levels were significantly different from unity, mainly for ICF and IFL. Table 4 lists the number of overlapping animals of the top 50 sires between extreme EGs. The more extreme between these EGs were, the lower the overlap of the top sires was, which indicates that the re-ranking of sires can happen across extreme EGs, especially for ICF.

**Table 3:** Genetic correlations (standard errors) across extreme EGs

Trait	*A*|*H*	Genetic correlation (1 and 99%)^a^	Genetic correlation (5 and 95%)^a^	Genetic correlation (10 and 90%)^a^	Genetic correlation (20 and 80%)^a^
AIS	***A***	0.773 (0.217)	0.883 (0.103)	0.932 (0.058)	0.971 (0.024)
	***H***	0.793 (0.189)	0.891 (0.092)	0.938 (0.050)	0.974 (0.021)
ICF	***A***	0.794* (0.105)	0.883* (0.063)	0.928* (0.039)	0.968* (0.018)
	***H***	0.793* (0.104)	0.882* (0.062)	0.928* (0.039)	0.968* (0.018)
IFL	***A***	0.901* (0.051)	0.952* (0.026)	0.970* (0.016)	0.987* (0.007)
	***H***	0.911* (0.047)	0.956* (0.023)	0.973* (0.015)	0.988* (0.006)
NRR	***A***	0.816 (0.172)	0.903 (0.096)	0.939 (0.060)	0.974 (0.026)
	***H***	0.818 (0.165)	0.903 (0.093)	0.940 (0.058)	0.975 (0.024)

*AIS* number of inseminations per conception, *ICF* interval from calving to first insemination, *IFL* interval from first to last insemination, *NRR* non-return rate at 56 days after first insemination, ***A*** and ***H*** pedigree-based and pedigree-genomic combined matrices, respectively

^a^The genetic correlations between the 1 and 99%, 5 and 95%, 10 and 90%, and 20 and 80% quantiles of EGs; one asterisk (*) means the value deviates from unity by more than 1.645 × SE.

**Table 4:** The number of overlapping animals of the top 50 sires along two extreme EGs

Trait	*A*|*H*	Top 50 overlaps (1 and 99%)^a^	Top 50 overlaps (5 and 95%)^a^	Top 50 overlaps (10 and 90%)^a^	Top 50 overlaps (20 and 80%)^a^
AIS	***A***	26	34	36	43
	***H***	24	32	37	42
ICF	***A***	23	26	29	37
	***H***	19	26	33	39
IFL	***A***	39	42	43	46
	***H***	38	41	43	45
NRR	***A***	37	40	42	44
	***H***	34	38	41	46

*AIS* number of inseminations per conception, *ICF* interval from calving to first insemination, *IFL* interval from first to last insemination, *NRR* non-return rate at 56 days after first insemination, ***A*** and ***H*** pedigree-based and pedigree-genomic combined matrices, respectively

^a^The number of overlapping animals in the top sires in the 1 and 99%, 5 and 95%, 10 and 90%, and 20 and 80% quantiles of EGs

### Accuracy of predicted breeding values

Table S2 lists the LRT statistics for the RM and RNM, which were all statistically significant, indicating the reasonability of the RNM. The accuracies of predicted breeding values for bulls in the validation dataset obtained by different models are listed in Table 5. The highest accuracy (0.319) was observed between GEBV and DYD for ICF using the RNM. The inclusion of genomic information improved the prediction accuracy in both the RM and RNM. The largest improvement was observed for AIS with the RM, which increased from 0.154 to 0.248. The accuracies for ICF and IFL obtained from the RNM were slightly higher than those obtained from the RM, and the largest improvement was observed in the EBV of IFL for the RNM, which increased from 0.133 to 0.140.

**Table 5:** The prediction accuracies for different traits using an RM and RNM

Trait	Model	Cor(EBV, DYD)^a^	Cor(GEBV, DYD)^b^
AIS	RM	0.154	0.248
	RNM	0.147	0.236
ICF	RM	0.262	0.318
	RNM	0.265	0.319
IFL	RM	0.133	0.218
	RNM	0.140	0.228
NRR	RM	0.123	0.127
	RNM	0.103	0.117

*AIS* number of inseminations per conception, *ICF* interval from calving to first insemination, *IFL* interval from first to last insemination, *NRR* non-return rate at 56 days after first insemination, *RM* reduced model, *RNM* reaction norm model

^a^The correlation coefficients between DYD and predicted breeding values estimated using the ***A*** matrix

^b^The correlation coefficients between DYD and predicted breeding values estimated using the ***H*** matrix

### ssGWAS for ICF and IFL

**Fig. 3:**
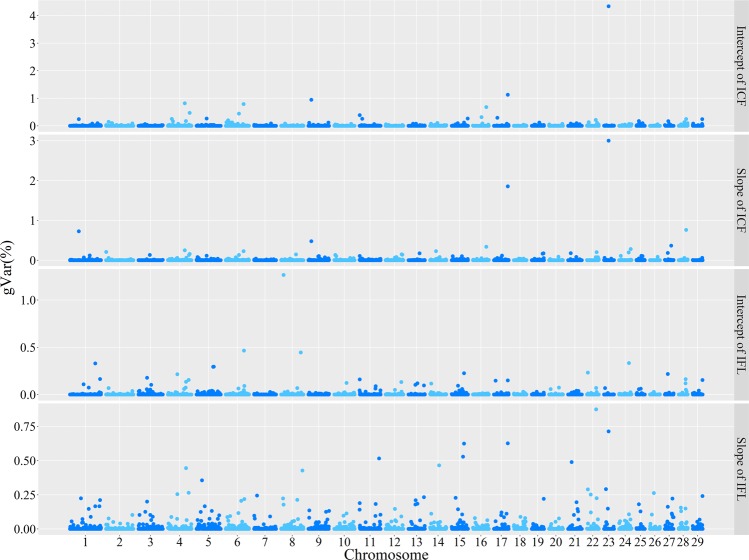
Proportions of the intercept and slope variances explained by each 20-SNP region for ICF (interval from calving to first insemination) and IFL (interval from first to last insemination) based on ssGWAS. The *x*-axis represents the chromosomes, and the *y*-axis shows the percentages of genetic variances

The percentages of genetic variances explained by genomic windows are shown in the Manhattan plot in Fig. 3. Table S3 shows the location information of top windows for the intercept, slope and candidate genes included in those windows. Some top windows that accounted for the largest variance of the intercept were also found to be the top windows that explained the largest variance of the slope for ICF (6 windows) and IFL (1 window), but the effects of these shared windows were mainly in opposite directions because of the negative correlation coefficients between the intercept and slope for ICF (−0.725), and for IFL (−0.360). The top genomic window explained 4.34% of the variance of the intercept for ICF, which also accounted for the largest percentage (3.00%) of the variance of the slope for ICF. Compared with ICF, the percentages of genetic variances explained by genomic windows for IFL were smaller. There were two genomic regions associated with both ICF and IFL, namely, 24.04–24.90 Mb on BTA23 and 70.78–71.59 Mb on BTA17. The candidate genes in these two windows were the immunity-related genes *IL17*, *IL17F* and *LIF*.

## Discussion

In this study, we explored the G × E interaction for female fertility traits using RNMs with or without genomic information and evaluated prediction accuracies for future performance. Further, we tried to locate the particular regions in the genome that probably explain the genetic background of ICF and IFL, which exhibited G × E interactions across extreme EGs. The results showed that the slope variances of the reaction norm for all the traits were statistically significant from zero, while genetic correlations across the extreme EGs could only be identified as significantly different from unity for ICF and IFL. Generally, the inclusion of genotype information improved the prediction accuracy. In addition, some candidate genes influencing intercepts and slopes of reaction norms for ICF and IFL were also identified.

### Heritabilities estimated from conventional linear mixed model

The heritabilities estimated from the conventional linear mixed model (Table 1) were similar to those from previous studies (Hou et al. 2009; Jamrozik et al. 2005; Liu et al. 2008, 2017). Several studies showed that heritability estimates for interval traits (IFL and ICF) were generally higher than those for count trait (AIS) and binary trait (NRR) for dairy cattle (Berry et al. 2013; Ghiasi et al. 2011), which was also true for our study.

### Variance components estimated with the RNM

In this study, we estimated variance components using an RNM with ***A*** or ***H*** matrices. A previous study on milk yield, dry matter and body weight of Holstein dairy cattle compared genetic variance estimates obtained from ssGBLUP with those obtained from conventional BLUP and showed that variance components estimated with the ***A*** matrix were higher than the components obtained from the ***H*** matrix, while the latter led to higher accuracy (Veerkamp et al. 2011). However, genetic variance estimates obtained from the conventional RNM and those from the genomic RNM were similar, which was consistent with the result of an earlier study on G × E interactions for total number born in pigs using an RNM (Silva et al. 2014).

Residual variances across different EGs could be different (Knap and Su 2008; Kolmodin et al. 2002). In our study, the heterogeneous residual variances were defined for different herd groups, but not at each herd level. This resulted in discontinuous estimates of heritability between herd groups, as shown in Fig. 1. We found that the residual variance components did not always increase along with the increase in herd effects for ICF and NRR. This pattern has also been observed and discussed in previous studies for G × E interactions in pigs (Silva et al. 2014) and cattle (Cardoso and Tempelman 2012). Calus et al. (2006) suggested that a higher-order RNM and alternative heteroskedastic error specifications might be used in analysis of G × E interactions.

### G × E interaction

In this study, the variance of the slope in the RNM was significantly different from zero for all traits, suggesting that all traits exhibited G × E interaction to some extent (Falconer 1990). The correlations between the intercept and slope for all the traits were strongly negative. Some studies asserted that a low correlation between the intercept and slope suggested the re-ranking of animals across different environments, which means that the best animal in one environment was not necessarily the best in another environment (Santana et al. 2013; Su et al. 2006). Actually, even if the correlation between the intercept and slope is very high, the re-ranking of animals can still occur as long as the range of EG is large enough and the variance of the slope is significantly different from zero, because the trajectories of different animals’ performances along the EG are not parallel. Therefore, the selection of robust animals with flat slopes and preferential intercepts in breeding practice is very important because they maintain the superior performance across different environments (Strandberg et al. 2000). In addition, environmental sensitivity must be taken into account in the selection of animals as it offers the opportunity to automatically include the environment in the breeding goal (De Jong and Bijma 2002). Furthermore, when RNM was used for a conventional breeding program, the breeding value for each herd level could be appropriate for selection of a heifer/cow, which is raised in a particular farm. However, the breeding value at average effect of herds could be appropriate for a bull, whose daughters are raised in different farms.

Conventional RNMs and genomic RNMs led to similar genetic correlations between different EGs for ICF and NRR (Fig. 2), which was consistent with previous estimates for yearling weight trait of beef cattle (Oliveira et al. 2018). However, slightly lower genetic correlation coefficients estimated by conventional RNMs were observed for AIS and IFL, which were consistent with previous results for total number born of pigs (Silva et al. 2014). As we have mentioned above, there existed differences of variance components estimated with different relationship matrices, but genomic relationship matrix led to more accurate estimates. Even so, the differences of estimates between genomic and conventional RNMs led to the similar results of existence of G × E interaction, i.e., the genetic correlations between extreme EGs were significantly from unity for ICF and IFL using both conventional and genomic RNMs (Table 3), indicating that the re-ranking of sires may occur if their daughters are distributed across different extreme EGs, which can be observed in Table 4. It was observed that re-ranking was more evident for ICF and AIS than for IFL and NRR (Table 4). Some previous studies have reported the existence of G × E interactions in different fertility traits for cows under different environmental descriptors. For instance, ICF was found to have a high sensitivity under different calving months in Danish and Swedish Holstein dairy cattle (Ismael et al. 2016). Significant G × E interactions in different herds of conventional and organic production systems were observed for calving interval, days open and pregnant at first insemination only in the second parity of Swedish Holsteins (Sundberg et al. 2010), but no G × E interactions existed in the first parity. This suggests that the existence of G × E interactions depends not only on the different environmental descriptors but also on the different stages within lactations.

### Accuracy of genomic prediction

Although the existence of G × E interactions between different EGs was not pervasive for all traits, the LRT results showed that the RNM fit the data better than the RM without considering G × E interactions of all these traits (Table S2). However, the LRT simply measures the goodness of fit of the model rather than the accuracy of prediction (Su et al. 2012). Therefore, the prediction accuracies were compared between the RM and RNM, and the RNM had slightly higher prediction accuracies for ICF and IFL, which is also consistent with the existence of G × E interactions across extreme EGs. Whether the accuracy of prediction for future performance could be improved by the inclusion of the reaction norm was examined by the evaluation of genomic predictions using the RNM with ***A*** and ***H*** matrices. Compared with pedigree-based BLUP, higher accuracies were obtained with the inclusion of genotype information. In a previous study that evaluated the genomic prediction accuracy using a conventional ssGBLUP model in the same Holstein population, the genomic prediction accuracy of ICF was evaluated to be 0.24 for pedigree-based BLUP and 0.29 for ssGBLUP (Ismael et al. 2017), which is comparable to the results we obtained in this study.

### Mapping of genomic regions associated with the intercept and slope of the reaction norm

This study performed ssGWAS to locate the particular regions along the genome that were associated with fertility phenotypes and environmental sensitivity. ICF and IFL were chosen to perform ssGWAS as significant G × E interactions were observed across extreme EGs for the two traits (Table 3), and slightly higher prediction accuracies for the RNM were observed compared to the RM (Table 5). Some identified genomic windows were common for both the intercept and slope for each of the two traits (Table S3), and more were found for ICF. The latter result could be because the absolute value of the correlation between the intercept and slope was high (0.725) for ICF, and low (0.360) for IFL. The overlaps of genomic windows between ICF and IFL contain some immune genes such as *IL17*, *IL17F* and *LIF*, which were proved to be related to fertility. For instance, *IL17* and *IL17F* encode interleukin 17 cytokine, and the increased level of this cytokine in plasma has a negative impact on human fertility (Ozkan et al. 2014). *LIF* encodes another interleukin cytokine, leukaemia inhibitory factor, which plays an important role in embryo implantation. It has been reported that aberrant leukaemia inhibitory factor production is linked to implantation failure (Salleh and Giribabu 2014). In addition, immunity-related genes were observed in top genomic windows for the intercept and slope of the two traits, such as *IL17*, *IL17F*, *IL17RB*, *LIF*, *CHDH*, *CD59* and *TLR4*, indicating influences of immunity on fertility in cattle. It has been reported that immunity and fertility could share some common elements (Hurley 2014). As different antimicrobials are used in different herds among different production systems (Bennedsgaard et al. 2006; Bennedsgaard et al. 2010), the pleiotropy of these immunity-related genes may play roles in the performance of fertility traits (Banos et al. 2013). Some genes in the top regions of the intercept and slope for ICF have been previously identified as contributing to cattle fertility. For instance, *TFG*, *EREG* and *AREG* are related to preovulatory follicles in cattle (Li et al. 2009), and *TPR* is linked to age at first calving in Nellore cattle (Mota et al. 2017). For IFL, the genomic regions influencing the intercept contain the *SMAD4* gene, which is necessary for early embryonic development and embryotrophic actions of follistatin in cattle (Lee et al. 2014). The genomic windows related to the slope for both traits also contain two growth-related genes, *MC4R* and *LEP*, both of which can regulate obesity and energy expenditure (Dempfle et al. 2004; Paolini et al. 2016; Rosenbaum and Leibel 2014; Rutanen et al. 2004). The association between these genes and ICF and IFL suggests that that fertility of cattle is an energy-dependent process, which could be influenced by another energy-consuming process of growth, based on the energy allocation theory (Heino 1999).

In conclusion, genetic parameters obtained from the RNM suggested the existence of G × E interactions for all traits, indicating that the breeding value of an individual may be changed in different herds for these traits. However, genetic correlations across extreme EGs indicated significant G × E interactions for ICF and IFL. The RNM resulted in a better goodness of fit than the RM for all the traits and higher prediction accuracies for ICF and IFL than the RM. Genotype information improved the prediction accuracies of both the RM and RNM for all of the traits. Several reproduction-related, immunity-related and growth-related genes were identified in the genomic regions affecting the intercepts and slopes of the reaction norm for ICF and IFL.

### Data archiving

Data supporting this paper were obtained from the commercial dairy farms in Denmark. The phenotype and genotype data are available only upon agreement with commercial breeding organizations and should be requested directly from the corresponding author.

## Supplementary information

Supplementary Information.
