# Supplementary material for: Genotype-by-environment interaction of fertility traits in Danish Holstein cattle using a single-step genomic reaction norm model
Source: Heredity (Edinb). 2019 Feb 13;123(2):202–14. doi: 10.1038/s41437-019-0192-4 (PMC6781120; doi:10.1038/s41437-019-0192-4)
Supplement: Supplementary file 1 — Supplementary Information. [file 41437_2019_192_MOESM1_ESM.pdf]

Table S1. The weights generated for each class of residual variance of each trait.

Trait[a]	Group	Residual variance	Weight
AIS	1	0.8713	1.6059
AIS	2	1.1525	1.2140
AIS	3	1.3736	1.0186
AIS	4	1.5898	0.8801
AIS	5	1.7686	0.7911
ICF	1	522.9747	1.3945
ICF	2	625.6533	1.1657
ICF	3	751.0613	0.9710
ICF	4	893.1548	0.8166
ICF	5	881.8061	0.8271
IFL	1	2383.8825	1.4226
IFL	2	2863.7357	1.1842
IFL	3	3384.3507	1.0021
IFL	4	3932.9976	0.8623
IFL	5	4547.3322	0.7458
NRR	1	0.2352	0.9984
NRR	2	0.2415	0.9724
NRR	3	0.2384	0.9850
NRR	4	0.2264	1.0373
NRR	5	0.1998	1.1755

[a] AIS: number of inseminations per conception; ICF: interval from calving to first insemination; IFL: interval from first to last insemination; NRR: non-return rate at 56 days after first insemination.

Table S2. Likelihood ratio test between RM and RNM.

Trait[a]	$\boldsymbol{A}\vert\boldsymbol{H}$[b]	likelihood ratio statistic (D)[c]	P-value[d]
AIS	$\boldsymbol{A}$	106.7	3.66E-24
	$\boldsymbol{H}$	105.7	6.01E-24
ICF	$\boldsymbol{A}$	114.4	7.79E-26
	$\boldsymbol{H}$	120.5	3.69E-27
IFL	$\boldsymbol{A}$	90.2	1.39E-20
	$\boldsymbol{H}$	91.2	8.59E-21
NRR	$\boldsymbol{A}$	7.65	1.37E-02
	$\boldsymbol{H}$	8.83	7.52E-03

[a] AIS: number of inseminations per conception; ICF: interval from calving to first insemination; IFL: interval from first to last insemination; NRR: non-return rate at 56 days after first insemination.

[b] $\boldsymbol{A}$ and $\boldsymbol{H}$: pedigree-based and pedigree-genomic combined matrices, respectively.

[c] The likelihood ratio statistic was calculated as D = -2 * log(likelihood) for the RM + 2 * log(likelihood) for the RNM.

[d] The P-value for the LRT was calculated as $0.5\mathrm{P}\left[\chi^2_{1\,d.f.} \geq \mathrm{D}\right] + 0.5\mathrm{P}\left[\chi^2_{2\,d.f.} \geq \mathrm{D}\right]$.

Table S3. Chromosomes (Chr), initial positions (Start), final positions (End), percentages of intercept and slope variances (gVar) explained by the top 10 genomic windows for ICF and IFL, and candidate genes located in these regions.

Trait	Intercept					Slope				
	Chr	Start	End	gVar	Genes	Chr	Start	End	gVar	Genes
ICF	23	24042335	24904300	4.34%	*IL17*;*IL17F*	23	24042335	24904300	3.00%	*IL17*;*IL17F*
	17	70776835	71585018	1.13%	*LIF*	17	70776835	71585018	1.85%	*LIF*
	9	14745626	15640590	0.94%		28	37682570	38961890	0.76%	
	4	86354358	87191167	0.82%		1	44752791	45614604	0.72%	*RAB5A*;*TFG*
	6	91023863	91906227	0.79%	*EREG*;*AREG*	9	14745626	15640590	0.48%	
	16	68014396	68889225	0.68%	*TPR*	27	32281266	33149699	0.37%	
	4	111183448	112080616	0.47%		16	68014396	68889225	0.34%	*TPR*
	6	67286349	68546212	0.44%		24	58983505	60208205	0.28%	*MC4R*
	11	1045889	1666202	0.39%		4	86354358	87191167	0.25%	
	16	43522152	45197527	0.32%	*NMNAT1*	6	91023863	91906227	0.23%	*EREG*;*AREG*

IFL	8	12067528	14030646	1.26%		22	47103229	48063014	0.87%	*IL17RB*;*CHDH*
	6	92922752	93961099	0.47%		23	24042335	24904300	0.71%	*IL17*;*IL17F*
	8	100308341	101231366	0.44%		17	70776835	71585018	0.63%	*LIF*
	24	50306117	51840480	0.33%	*MBD1*;*SMAD4*	15	64436323	65408389	0.62%	*CD59*
	1	128962989	129774934	0.33%		15	59183752	60144021	0.53%	*BDNF*
	5	87843519	88725393	0.29%		11	99096532	100108508	0.52%	
	5	84941287	86142655	0.29%	*BCAT1*	21	18338067	19379299	0.49%	
	22	4735174	5546862	0.23%	*TGFBR2*	14	47298921	48226460	0.46%	*TNFRSF11B*
	15	64436323	65408389	0.23%	*CD59*	4	92742789	93739544	0.44%	*LEP*
	27	14671414	15447004	0.22%		8	108556349	109347548	0.43%	*TLR4*

[a] ICF: interval from calving to first insemination; IFL: interval from first to last insemination.
